# The Toxic Factor in Certain Mental Diseases

**Published:** 1907-02

**Authors:** 


### THE TOXIC FACTOR IN CERTAIN MENTAL DISEASES.

By Dr. Rebizzi (Rivista di patologia nervosa ementale, XI., 1906).

The author studied the toxicity of the blood in various diseases of the mind, by means of a very ingenious method. A number of leeches were permitted to fill themselves with the blood of the patients, and at the end of twenty-four hours the nervous system of the animals was examined in regard to changes at the intracellular fibrils of the ganglia. These structures presented a well-marked atrophy in those cases where the leeches had imbibed the blood of epiletics. Similar pictures were obtained in paralysis and in senile dementia, whereas in amentia there was some hypertrophy of the fibrils, with fragmentation and granular disintegration. The same findings were noted in the confusional states of patients having pellagra. The nervous system of the leeches remained normal in cases of alcoholic psychosis, in idiocy, and in dementia precox. The author concludes from these experiments that those diseases which yielded positive findings are based upon the presence of poison with a specific action on the nervous system. Alcoholic psychoses and dementia precox are presumably likewise of toxic origin, but the poison can no longer be demonstrated in the later stages of these diseases.—The Post-Graduate, December, 1906.

